# Selection and Characterization of a Nanobody Biosensor of GTP-Bound RHO Activities

**DOI:** 10.3390/antib8010008

**Published:** 2019-01-09

**Authors:** Laura Keller, Nicolas Bery, Claudine Tardy, Laetitia Ligat, Gilles Favre, Terence H. Rabbitts, Aurélien Olichon

**Affiliations:** 1 Centre de Recherche en Cancérologie de Toulouse (CRCT), Inserm, Université Paul Sabatier-Toulouse III, CNRS, 31037 Toulouse, France; keller.laura@iuct-oncopole.fr (L.K.); claudine.tardy@inserm.fr (C.T.); laetitia.ligat@inserm.fr (L.L.); favre.gilles@iuct-oncopole.fr (G.F.); 2 Institut Claudius Regaud (ICR), Institut Universitaire du Cancer de Toulouse-Oncopole (IUCT-O), Laboratoire de Biologie Médicale Oncologique (LBMO), 31059 Toulouse, France; 3 Weatherall Institute of Molecular Medicine, MRC Molecular Haematology Unit, University of Oxford, Oxford OX3 9DS, UK; nicolas.bery@ndcls.ox.ac.uk (N.B.); terence.rabbitts@imm.ox.ac.uk (T.H.R.)

**Keywords:** nanobody, phage display, intrabody, intracellular antibody, GTPase RHO, BRET, RAS

## Abstract

RHO (Ras HOmologous) GTPases are molecular switches that activate, in their state bound to Guanosine triphosphate (GTP), key signaling pathways, which involve actin cytoskeleton dynamics. Previously, we selected the nanobody RH12, from a synthetic phage display library, which binds the GTP-bound active conformation of RHOA (Ras Homologous family member A). However, when expressed as an intracellular antibody, its blocking effect on RHO signaling led to a loss of actin fibers, which in turn affected cell shape and cell survival. Here, in order to engineer an intracellular biosensor of RHOA-GTP activation, we screened the same phage nanobody library and identified another RHO-GTP selective intracellular nanobody, but with no apparent toxicity. The recombinant RH57 nanobody displays high affinity towards GTP-bound RHOA/B/C subgroup of small GTPases in vitro. Intracellular expression of the RH57 allowed selective co-precipitation with the GTP-bound state of the endogenous RHOA subfamily. When expressed as a fluorescent fusion protein, the chromobody GFP-RH57 was localized to the inner plasma membrane upon stimulation of the activation of endogenous RHO. Finally, the RH57 nanobody was used to establish a BRET-based biosensor (Bioluminescence Resonance Energy Transfer) of RHO activation. The dynamic range of the BRET signal could potentially offer new opportunities to develop cell-based screening of RHOA subfamily activation modulators.

## 1. Introduction

The RAS HOmologous RHO GTPases are small G proteins that act as molecular switches. These GTPases cycle between two conformational states depending on their binding to GDP (Guanosine diphosphate) or GTP (Guanosine triphosphate). A plethora of guanine nucleotide exchange factors (GEF) can stimulate the GTP loading of the small GTPases in a spatio-temporal manner, leading to local activation of effector proteins. A number of GTPase-activating proteins (GAP) can then catalyze the nucleotide hydrolysis to switch off the signaling [1]. Like for RAS, a subtle conformational change involving the two switch loops in the GTP-bound conformation of RHO enables the binding of the signaling effectors [2]. In contrast to the GTPase RAS-GTP, which can be massively induced [3], co-precipitation of the RHO-GTP pool by recombinant effector binding domains (RHO binding domain, RBD) showed that only a small fraction of the total RHO GTPases cellular pool is stimulated [4]. The RHOA-like subfamily includes RHOA/B/C members, which have been extensively studied for their involvement in actin cytoskeleton dynamics regulation, in cell proliferation and migration, or in development [5]. According to the physiological context, they can drive several other key signaling pathways such as YAP (Yes associated protein) for RHOA [6,7] or AKT for RHOB (RAS Homologous family member B) [8,9]. Their potential involvement in cancer cell migration and metastasis [5,10,11], or in targeted therapy resistance [9,12], suggests that the development of small molecule inhibitors targeting these GTPases would be valuable. For example, the small molecule RHOSIN inhibits the interaction of RHO with several GEFs (LARG, DBL, LBC, p115-RHOGEF, PDZ-RHOGEF), but this inhibitor presents limited efficiency in most cellular contexts [13].

The difficulty in identifying potent cellular inhibitors of RHO is partly the result of the lack of quantitative tools to precisely monitor their cellular activation. To date, all the molecular tools available to study RHO activity states are based on the use of effector RBDs either in pull down or capture ELISA (Enzyme-linked Immunosorbent assay) assays on cellular lysates, as intramolecular FRET-based sensors (Fluorescent probes based on Förster Resonance Energy Transfer) [14,15], or as tripartite split-GFP protein–protein interaction reporters in cells [16]. However, the poor stability of the RBD, as well as its low affinity towards the GTP-bound RHOs, has hampered the dynamic range of these assays [17,18]. In addition, the use of such effector domains in cells could be per se a potential competitor of the endogenous effectors, thus these tools require optimization of expression level in stable cell lines. Therefore, artificial affinity binding domains with higher stability and selectivity offer an attractive alternative to develop biosensors of RHO activation.

Nanobodies or stable single domain antibodies have emerged as useful molecular reagents to sense or track antigens in the reducing intracellular environment when used as intracellular antibodies [19,20]. Their high solubility in the reducing cytosol retains their conformational specificity and high affinity required for the selective recognition of antigens in living cells. We and others have already reported such alternative binding domains selective towards the GTP-bound conformation of a GTPase such as H-RAS [21], Dynamin [22], or RHO/RAC subfamilies [23]. In particular, we identified a synthetic nanobody (designated RH12) with high affinity towards the RHOA-like subfamily and RAC1 proteins, and with high specificity to their GTP-bound form in vitro and in cells. When expressed as an intracellular antibody, RH12 nanobody induced a dramatic effect on cell shape that was associated with actin polymerization defects [23]. We assumed that the RH12 nanobody acts as a macrodrug by blocking GTP-bound RHO and RAC signaling and inhibiting the RHO-RAC/effector interactions. Therefore, such a blocking intracellular antibody would not be suitable to build a biosensor. 

Here, we report the characterization of another high affinity nanobody (referred to hereafter as RH57) that is specific to the GTP-bound fraction of RHOA and RAC1 subfamilies in vitro, but with no apparent competition with RHOA-like effectors when used as an intracellular antibody. We expressed it as a chromobody and observed its re-localization to the plasma membrane upon RHO stimulation. We functionalized the nanobody in order to produce a versatile dynamic BRET biosensor of RHO activation in cells. This clone not only provides an additional example of the diversity and functionality of the synthetic nanobody phage library from which it has been generated, but also illustrates the ability of intracellular antibodies to study the activity of challenging proteins.

## 2. Material and Methods

### 2.1. Plasmids

p-IB-6His-Myc and pIB-GFP intracellular antibodies and chromobodies expression vectors, in which any humanized synthetic single domain antibody (hs2dAb) from the NaLi-H1 library can be inserted by NcoI and NotI cloning sites, were described previously [23]. The 2x-Strep-tag^®^ (similar to Twin-Strep-tag® from IBA-Lifesciences®) followed by HA tag (2SHA)-RHO plasmid expression was described previously [23]. pHEN-hs2dAb 6xhis-myc-6xhis was constructed as following: hs2dAb-6xhis-myc was digested from pIB-GFP and inserted into a modified pHEN6-VHH-6xhis that was previously described [24], thus creating a periplasmic expression vector pHEN6-hs2dAb-6xhis-myc-6xhis. The tag downstream of the NotI site was replaced by a synthetic Flag-Ctag DNA fragment encoding the following translated sequence: A A A G G G S G G D Y K D D D D K G Y Q D Y E P E A *, thus producing pHEN-hs2dAb-Flag-Ctag. The hs2dAb-Flag-Ctag was then sub-cloned into the previously described pAOT7 vector [23] using NcoI and EcoRI sites to produce the pAOT7-hs2dAb-Flag-Ctag, which allowed the production in *E.coli* cytoplasm.

### 2.2. Cell Lines, Transfection Method, and Reagents

HeLa cells (cervix adenocarcinoma; ATCC^®^ CRM-CCL-2™, ATCC, Manassas USA) were grown in Dulbecco’s Modified Eagle Medium (DMEM) (Lonza^®^, Basel, Switzerland) supplemented with 10% FBS (Foetal Bovine Serum) (Sigma Aldrich^®^, St. Louis, MO, USA). Transient transfections of DNA plasmids were performed using the Jet Prime method, as indicated by the supplier (PolyPlus Transfection^®^, Illkirch, France).

HEK293T human embryonic kidney cells (ATCC^®^ CRL-3216) were grown in DMEM medium (Life Technologies^®^, Carlsbad, CA, USA) supplemented with 10% FBS (Sigma Aldrich^®^) and 1% Penicillin/Streptomycin (Life Technologies^®^). HEK293T cells were transfected with Lipofectamine 2000 (Thermo-Fisher^®^, Waltham, MA, USA, see BRET2 section). All cells were grown at 37 °C in a humidified incubator with 5% CO_2_.

Western blots were probed with a mouse monoclonal 26C4 anti-RHOA (1/500, O/N, 4 °C, Santa Cruz Biotechnology^®^, Dallas, TX, USA), goat polyclonal anti-myc tag HRP conjugated (1/3000, 1 h, room temperature (RT), Novus Biologicals^®^, Centennial, CO, USA), and mouse monoclonal anti-RAC1 (1/1000, O/N, 4°C, Millipore). Detection was performed using peroxidase-conjugated secondary antibodies and a chemiluminescence detection kit (Biorad^®^, Hercules, CA, USA). F-Actin was stained with Alexa568-conjugated phalloidin (Molecular Probes, Eugene, USA). 

### 2.3. Subtractive Phage Display Panning for Isolating RHO-GTP Specific hs2dAb

**Figure 1:**
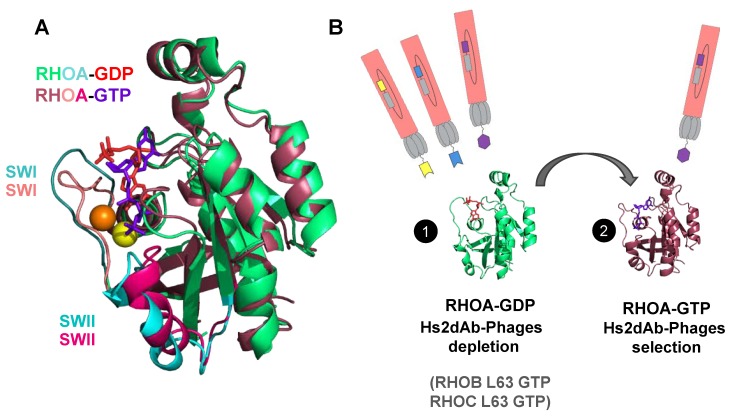
Antibody phage display selection of GTP-bound RHO conformational nanobodies. (**A**) View of the structure of RHOA-GTP V14 mutant (shown in wine-red and pink, Protein Data Base (PDB): 1a2b) superimposed with the structure of RHOA-GDP (shown in green and cyan, PDB: 1ftn). RHOA G14V mutant in the active state is bound to the GTP (purple-blue nucleotide) and Mg^2+^ (shown as a yellow sphere). RHOA displays the inactive conformation bound to GDP (red nucleotide) and Mg^2+^ (shown as an orange sphere). The structural alignment in this view shows a subtle closure of the switch I and switch II loops (SWI and SWII loops currently in cyan in RHOA-GDP and in pink in RHOA-GTP) around the phosphate gamma and the Mg^2+^ (orange to yellow). (**B**) Scheme of the subtractive phage display enrichment of hs2dAb to GTP-bound RHOA (wine-red) by depletion with the inactive GDP-bound state (green) and with the GTP-bound state of RHOB and RHOC (see Methods). The phages presenting hs2dAb are shown in pink/grey. The RHOA structures were produced using PyMOL software 2.1 (Schrödinger, Mannheim, Germany).

The NaLi-H1 library of humanized synthetic single domain antibody [23] was used for this study. A subtractive panning protocol was designed to isolate hs2dAb selective for RHOA-GTP conformation. The chitin binding domain (CBD) from chitinase A1 (New England Biolabs^®^, Ipswich, USA) or 2x-Strep-tag^®^ (IBA-Lifesciences^®^, Göttingen, Germany) fusion of RHOA GTPase active mutant (RHOA L63) were expressed transiently for 24 h in HEK293 cells and captured freshly after cell lysis on magnetic beads before incubation with the library phages. Chitin magnetic beads (New England Biolabs^®^) or Strep-Tactin^®^-coated beads (IBA-Lifesciences^®^) were used alternatively for the capture of antigens for the four rounds of phage display. Phages were previously adsorbed on empty chitin or Strep-Tactin^®^-coated magnetic beads to remove nonspecific binders. From the second round of panning, depletion steps on GDP-loaded wild type RHOA or N19 inactive mutant and on RHOB L63, RHOC L63, and RAC1 L61 active mutants were included (Figure 1B). The adequate amount of antigen-coated beads was incubated for 2 h with the phage library (10^13^ phages diluted in 1 mL of PBS + 0.1% Tween 20 + 2% non-fat milk). Phages and antigens-bound Strep-Tactin^®^-coated beads or Chitin beads were recovered on a magnet. Beads were washed with PBS–Tween 0.1% 10 times (round 1), 15 times (round 2), or overnight (rounds 3 and 4), and in the presence of an excess of untagged RHOA and RHOC L63 to further deplete in binders with a high dissociation rate. Bound phages were eluted using triethylamine (Sigma Aldrich^®^) and *E. coli* (TG1 strain) were infected with the eluted phages. For rounds 2, 3, and 4, only 10^12^ phages were used as input.

### 2.4. Hs2dAb Purification

**Figure 2:**
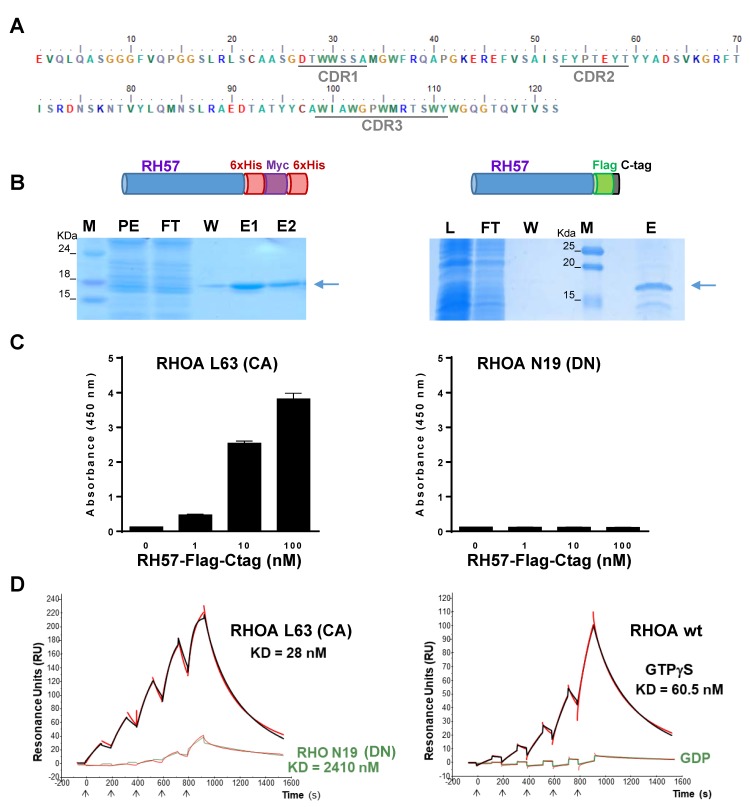
In vitro characterization of recombinant RH57 hs2dAb. (**A**) Amino acid sequence of the RH57 hs2dAb. Complementary-determining region (CDR) are underlined. (**B**) Representative Coomassie staining of SDS-PAGE showing the purification of RH57 fused to its carboxy terminal end with 6xHis-Myc-6xHis tag expressed in the periplasm (left) or RH57 fused to its carboxy terminal end with Flag tag and C-tag (right) used in the study. PE = periplasmic extract, L = clarified lysate, FT = flow through, W = wash, E = elution, and M = molecular weight marker. **(C)** ELISA detection of recombinant RHOA L63 constitutively active (CA) mutant versus RHO N19 dominant negative (DN) mutant. RHO proteins were captured on a Strep-Tactin coated plate and incubated with increasing concentrations of recombinant RH57-Flag-Ctag. Anti-Flag antibody was used to reveal the bound fraction of recombinant RH57 hs2dAb. **(D)** Single cycle kinetics (SCK) surface plasmon resonance (SPR) measurements on RH57-6xhis-myc-6xhis captured on an Ni-NTA (Nickel affinity capture of 6xHis) chip. Red lines correspond to the raw data measurements, and the fit curves are displayed in black or green according to the condition. Analytes were recombinant RHOA L63 CA mutant (black line, left SCK); RHOA N19 DN mutant (green line, left SCK); or RHOA WT loaded with either GTPγS (black line, right SCK) or GDP (green line, right SCK), injected at increasing concentrations (arrows, see Methods).

Briefly, hs2dAb-6 × His-Myc-6 × His was produced in the periplasm of XL1BLUE *E. coli* grown in TB/ampicillin (100 μg/mL) medium supplemented with 1% glucose in the start culture and 0.5% glucose during induction with 1 mM IPTG (Isopropyl β-D-1-thiogalactopyranoside). After overexpression for 16 h at 28 °C, the cells were harvested, suspended in 15 mL ice-cold TES buffer (200 mM Tris (Sigma Aldrich^®^) pH 8.5, 0.5 mM EDTA, 500 mM Sucrose), and stored at −80°C. Thirty milliliters of a one-quarter dilution of TES buffer was briefly added to the re-suspended pellets prior to vortex and kept at 4 °C for 30 min. After centrifugation (30 min, 13000× *g*, 4 °C), the periplasmic extract containing hs2dAb was purified by affinity chromatography. The protein extract was incubated for 2 h in the presence of complete His-Tag purification beads (ROCHE^®^, Basel, Switzerland) previously equilibrated with an equilibration buffer (50 mM Tris pH 8.0, 0.125 mM EDTA, 125 mM sucrose, 100 mM NaCl, 10 mM imidazole pH 7.0). The beads were washed with 30 mL of washing buffer (10 mM Tris pH 8.0, 150 mM NaCl, 10 mM imidazole pH 7.0). Hs2dAb were then eluted with elution buffer (500 mM imidazole pH 7.0, 25 mM Tris pH 6.8, 300 mM NaCl) and dialysed against PBS containing 20% glycerol for 16 h at 4 °C, and purity was assessed by SDS-PAGE followed by InstantBlue^TM^ (Expedeon, Cambridgeshire, UK) Coomassie staining (Figure 2B).

Cytosolic expression of hs2dAb-Flag-Ctag was performed in BL21(DE3) *E.coli* cells from the pAOT7 vector. Transformed bacteria cells were used to grow 3 mL TB/kanamycin (35 μg/mL) cultures overnight at 37 °C prior to dilution of the pre-culture in baffled flasks containing 1 L of the same media. Cells were allowed to grow at 37 °C until OD600 reached 0.5 to 0.7. Cells were then induced with IPTG at a final concentration of 100 μM and grown for an additional 16 h at 18 °C. Cells were harvested by centrifugation at 4000× *g* for 20 min. The pellets were re-suspended in lysis buffer (PBS pH 7.4, 1X lysozyme and DNase I, proteases inhibitors) and lysed by sonication on ice prior to centrifugation (30 min, 15,000× *g*, 4 °C). A CaptureSelect C-tag affinity matrix (Thermo-fisher^®^) was equilibrated in PBS pH 7.4. After loading clarified lysate and washing in PBS pH 7.4, 300 mM NaCl, 20 mM MgCl_2_, C-tagged antibodies were eluted in 20 mM Tris pH 7.4 in the presence of 2 M MgCl_2_. Dialysis was performed overnight against PBS containing 20% glycerol, and purity was assessed by SDS-PAGE followed by InstantBlue^TM^ (Expedeon, Cambridgeshire, UK) Coomassie staining (Figure 2B).

### 2.5. RHO GTPases Purification

RHO proteins deleted for the carboxy terminal four amino acids referred to as CAAX motif were expressed with an amino terminal tag that consisted of 2x-Strep-tag followed by HA tag (2SHA). 2SHA-RHO were expressed in BL21(DE3) *E.coli* cells from a pET vector. Transformed bacteria cells were used to grow 3 mL LB/carbenicillin (100 μg/mL) cultures overnight at 37 °C prior to inoculation in baffled flasks containing 1 L of the same media. Cells were allowed to grow at 37 °C until OD600 reached 0.5 to 0.7. Cells were then induced with IPTG at a final concentration of 100 μM and grown for an additional 20 h at 25 °C. Cells were harvested by centrifugation at 4000× *g* for 20 min. The pellets were re-suspended in lysis buffer (50 mM Tris HCl pH 8.0, 150 mM NaCl, 5 mM MgCl_2_, 0.1% triton, 1 mM DTT, 1X lysozyme and DNase I, proteases inhibitors) and lysed by sonication on ice prior to centrifugation (15 min, 10,000× *g*, 4 °C). Strep-Tactin SuperFlow Plus (IBA-Lifesciences ^®^) matrix was equilibrated in buffer A (50 mM Tris HCl pH 8.0, 150 mM NaCl, 5 mM MgCl_2_) and was incubated with supernatant for 2 h at 4 °C. Then, the supernatant and matrix were loaded on a simple column in order to maximize the capture of 2SHA-RHO proteins. The matrix was washed by 15 mL of washing buffer (300 mM NaCl, 50 mM Tris pH 8.0, 5 mM MgCl_2_, 0.1% Tween20). RHO proteins were then eluted in buffer A containing 10 mM Biotin (Sigma Aldrich^®^). Dialysis was performed overnight against buffer A containing 15% glycerol.

### 2.6. ELISA Assays

Wells of Strep-Tactin-coated plates (IBA-Lifesciences^®^) were coated with 100 nM of recombinant 2S-HA-tagged RHOA protein (200 μL in TBS by well) during 2 h at RT and then blocked with 5% milk in TBS–Tween 0.05% (blocking buffer) for 1 h at RT. Several dilutions of hs2dAb in blocking buffer were applied to the ELISA plates in duplicates for 1 h at RT. Next, we added 0.5 μg/mL anti-Flag HRP antibody (F1804, Sigma-Aldrich^®^) in blocking buffer for 1 h at RT and the reaction was visualized by the addition of 100 μL chromogenic substrate (Thermoscientific^®^, 1-step ultraTMB) for 3 min. The reaction was stopped with 50 μL H_2_SO_4_ 1N and absorbance at 450 nm was measured using a FLUOstar OPTIMA microplate reader (BMG LABTECH, Ortenberg, Germany). Plates were washed three times with washing buffer (TBS–Tween20 0.05%) after each step. All steps were performed under agitation (400 rpm).

### 2.7. Loading Recombinant Proteins with GTPγS/GDP

We added 10 mM EDTA and 1 mM GDP or 100 μM GTPγS to recombinant proteins 2S-HA-RHOA wt. We incubated the reaction for at least 30 min at 30 °C for recombinant proteins. The reaction was stopped by adding 60 mM MgCl_2_, and the mix was vortexed and put on ice.

### 2.8. Immunofluorescence Staining

Cells transfected with intracellular antibodies fused to a monomeric GFP (GFP carrying mutation Cys48Val) were fixed in 3.7% paraformaldehyde and permeabilized with PBS–Triton 0.1%, blocked with PBS–BSA 8%. Actin fibers were stained with Alexa 568-Phalloidin (1/40, 1 h, RT, Invitrogen^®^). All coverslips were mounted in Mowiol. Data acquisition was carried out on a Zeiss axiovert inverted microscope and analysed using Image J software (Fiji open-source platform).

### 2.9. Endogenous RHO Proteins Intracellular Antibodies Co-Immunoprecipitation Assays

**Figure 3:**
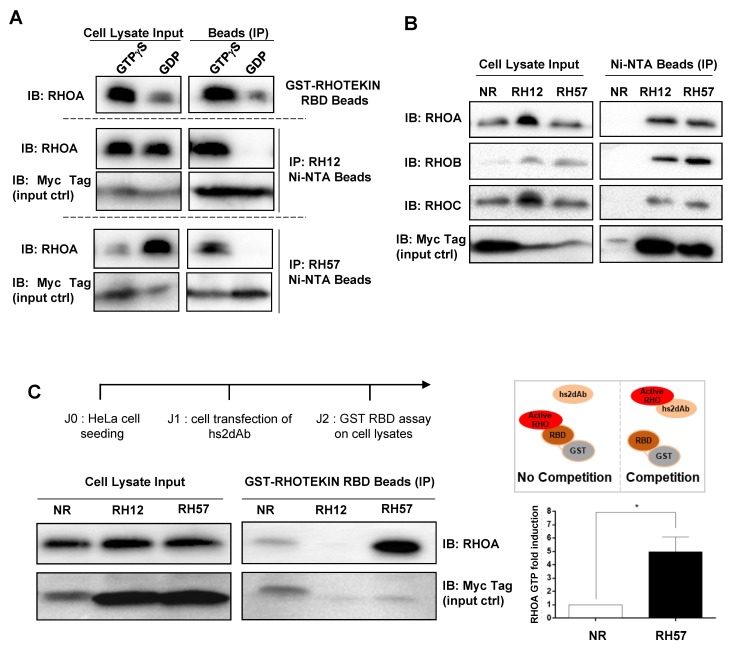
Intracellular antibody characterization of RH57 hs2dAb. (**A**) The hs2dabs RH12 and RH57 were expressed in HeLa cells with a 6xhis and carboxy Myc tag and the endogenous RHO GTPases were loaded with either GTPγS or GDP in the cell lysate. The intracellular antibodies were immuno-precipitated with Capture Select His tag affinity beads and an anti-RHOA antibody was used to monitor which pool of RHO (GTP or GDP) was bound by the intracellular antibodies. The recovered intracellular antibodies were monitored by immuno-blotting with anti-Myc tag. The GST-RHOTEKIN RHO binding domain (RBD), which allows to pull down GTP-bound RHO proteins on glutathione beads, was used as positive control. (**B**) Intracellular antibody immuno-precipitation assays on endogenous RHO proteins. Assays were performed as in panel A, but on non-loaded endogenous RHO proteins. The non-related NR hs2dAb was used as a negative control. **(C)** GST-RHOTEKIN RBD interference assay intracellular antibodies. The hs2dabs NR, RH12, and RH57 were expressed in HeLa cells with a 6xHis and carboxy Myc-tag. Intracellular expression of RH12 impedes the GST-RBD capture of RHOA-GTP. RH57 induced an increase of RHOA-GTP. NR binds to the beads. Representative blot of three independent experiments. Right: scheme of the binding competition events. Quantification of RHOA-GTP fold increase in RH57 conditions compared with the NR condition reveals potential stabilization of RHOA-GTP or GAP inhibition activity by the RH57. Error bars are mean ± SD of the biological repeats. * *p* < 0.05. IP = immuno-precipitation; IB = immuno-blot.

The hs2dabs RH12, RH57, or NR with a 6xhis and carboxy Myc tags were transiently expressed in HeLa cells. After 24 h, cells were harvested and lysed in buffer (50 mM Tris pH 7.4, 500 mM NaCl, 10 mM MgCl_2_, 1% TritonX100, supplemented with proteases and phosphatases inhibitors). To load the GTPases (Figure 3A), 10 mM EDTA and 1 mM GDP or 100 μM GTPγS were then added to cell lysates, and incubated for at least 30 min at 37 °C. The reaction was stopped by adding 60 mM MgCl_2_, and the mix was vortexed and put on ice. To pull down the intracellular antibody from the cell lysate, the loaded lysate or the fresh lysate (in Figure 3A,B, respectively) were incubated on Complete His-Tag Ni^2+^-NTA Purification Resin (ROCHE^®^) that had previously been equilibrated in the lysis buffer. After one hour at 4 °C, the beads were washed three times in buffer (50 mM Tris-HCl pH 7.4, 150 mM NaCl, 10 mM MgCl_2_, 0.1% Tween20) and denatured in 1X Laemmli reducing sample buffer, boiled for 5 min, and separated on 12.5% SDS-PAGE for Western blot analysis.

### 2.10. GST-RBD Assay

The hs2dabs RH12, RH57, or NR with a 6xhis and carboxy Myc tags were transiently expressed in HeLa cells. After 24 h, cells were harvested and lysed on ice in buffer (50 mM Tris-HCl pH 7.4, 500 mM NaCl, 10 mM MgCl_2_, 1% TritonX100, supplemented with proteases and phosphatases inhibitors). To pull down the GTP-bound RHOA/B/C with the reference method, the lysates were incubated with GST-RBD beads (Figure 3A,C). After 45 min at 4 °C, the beads were washed three times in buffer (50 mM Tris-HCl pH 7.4, 150 mM NaCl, 10 mM MgCl_2_, 0.1% Tween20) and denatured in 1X Laemmli reducing sample buffer, boiled for 5 min, and separated on 12.5% SDS-PAGE for Western blot analysis. Quantification of the RHOA immunoblot signal was done with ImageLab^TM^ version 6.0 (Biorad^®^, Hercules, CA, USA) software for each condition to determine the ratio of RHOA-GTP/total RHOA. The results for the RH57 condition were normalized to NR. Statistical analysis (*t*-test) was calculated using GraphPad Prism software version 6.0 (California corporation, USA) * for *p* < 0.05 (Figure 3C).

### 2.11. Affinity Measurement

Hs2dAb binding studies based on SPR (surface plasmon resonance) technology were performed on a BIAcore T200 optical biosensor instrument (GE Healthcare^®^, Uppsala, Sweden). Capture of recombinant hs2dAb-6xHis was performed on a nitrilotriacetic acid (NTA) sensor chip in HBS-P + buffer (10 mM HEPES pH 7.4, 150 mM NaCl, and 0.05% surfactant P20) (GE Healthcare^®^). The four flow cells (Fc) of the sensor chip were used: one (Fc 1) to monitor nonspecific binding and to provide background corrections for analyses and the other three flow cells (Fc 2, 3, and 4) containing immobilized hs2dAb-6xHis for measurement.

For immobilization strategies, flow cells were loaded with nickel solution (Sigma Aldrich^®^) (10 μL/min for 60 s) in order to saturate the NTA surface with Ni^2+^ and an extra wash using running buffer containing 3 mM EDTA after the nickel injection. His-tagged hs2dAb in running buffer was injected in flow cells at a flow-rate of 10 μL/min. The total amount of immobilized hs2dAb-6xHis was 250–300 resonance units (RUs; 1 RU corresponds to approximately 1 pg/mm^2^ of protein on the sensor chip). A single-cycle kinetics (SCK) analysis to determine association, dissociation, and affinity constants (ka, kd, and K_D_, respectively) was carried out. The SCK method prevents potential inaccuracy due to sensor chip regeneration between cycles, which is necessary in the conventional multiple cycle kinetics (MCK). The SCK binding parameters are evaluated for each injection according to the tools and fit models of the BIAevaluation software, giving similar values to MCK. As hs2dAb were smaller proteins than their respective antigens, hs2dAb were captured on the sensor chip, then the recombinant antigens were used as analyte and were injected sequentially with two-fold increased concentrations in a single cycle without regeneration of the censorship between injections. Concentrations of recombinant 2S-HA-RHOA L63 or N19 mutants ranged between 3.125 nM and 50 nM, and concentrations of 2S-HA-RHOA WT loaded with GDP or GTPγS ranged between 12.5 nM and 200 nM. Binding parameters were obtained by fitting the overlaid sensorgrams with 1:1. Langmuir binding model of the BIAevaluation software version 1.0 (GE Healthcare^®^, Uppsala, Sweden).

### 2.12. BRET

#### 2.12.1. Molecular Cloning

RHOA WT, RHOA L63, RHOB L63, RHOC L63, and RHOA N19 were cloned between NotI/XbaI of the pEF-RLuc8-(GGGGS)1-MCS described elsewhere [25]. The RH57 nanobody was inserted between NcoI/XhoI into pEF-GFP^2^-(GGGGS)1-MCS [25]. 

#### 2.12.2. BRET2 Titration Curves and Stimulation Assays 

For all BRET experiments (titration curves and stimulation assays), 650,000 HEK293T were seeded in each well of six-well plates. After 24 h at 37 °C, cells were transfected with a total of 1.6 g of DNA mix, containing the donor (RLuc8 plasmid) + acceptor (GFP^2^ plasmid) using Lipofectamine 2000 transfection reagent (Thermo-Fisher). Cells were detached 24 h later, washed with PBS, and seeded in a white 96-well plate (clear bottom, PerkinElmer) in OptiMEM without phenol red medium complemented with 4% FBS or no FBS for the stimulation assays. Cells were incubated for an additional 20–24 h at 37 °C before the BRET assay readings. Cells were stimulated for 12 min with 20% serum (20 L of 100% serum added in 80 L of BRET medium without FBS) before the BRET readings. 

#### 2.12.3. BRET2 Measurements

The BRET2 signal was determined immediately after addition of coelenterazine 400a substrate (10 M final) to cells (Cayman Chemicals), using a CLARIOstar instrument (BMG Labtech) including the luminescence module (filter settings: 515 nm–30 nm and 410 nm–80 nm). Total GFP^2^ fluorescence was detected with excitation and emission peaks set at 405 nm and 515 nm respectively. Total RLuc8 luminescence was measured with the filter set up at 410 nm–80 nm. 

The BRET signal (or BRET ratio) corresponds to the light emitted by the GFP^2^ acceptor constructs (515 nm–30 nm) upon addition of coelenterazine 400a divided by the light emitted by the RLuc8 donor constructs (410 nm–80 nm). The background signal is subtracted from that BRET ratio using the donor-only negative control, where only the RLuc8 plasmid is transfected into the cells. Total GFP^2^ and RLuc8 signals were used to control the protein expression level from each plasmid.

## 3. Results

### 3.1. Phage Display Selection of a New High Affinity GTP-Bound RHO Conformational hs2dAb

We previously selected, by phage display, the RH12 (hs2dAb from the synthetic nanobody library NaLi-H1 [23]. The RH12 pan-RHO-GTP blocking intracellular antibody was the only specific clone after five rounds of phage display selection and enrichment cycles. Albeit, RHOA conformational change according to its bound nucleotide involves only a small interface that comprises the switch domains (SWI and SWII, Figure 1A), we hypothesize that several other epitopes can be targeted by affinity reagents as small as nanobodies for an alternative selective recognition of the small GTPase active state. In order to obtain other RHO-GTP conformational nanobodies with different properties than those of the RH12, we slightly modified our subtractive phage display selection protocol that included depletion steps on an excess of inactive GDP-bound RHOA before capture on the active RHOA bound to non-hydrolysable GTPγS (Figure 1B). First, we reduced the number of washing steps in the first two rounds of panning to initially select more diversity of binding domains, then we increased the washing time in the third and fourth rounds up to overnight to keep high affinity nanobodies. We also increased the excess of competitors RHOA-GDP or the active mutant of the closest RHO (RHOB L63, RHOC L63, and RAC1 L61). After phage and fragment ELISA preliminary screening of monoclonal hs2dAb on both conformations of RHOA, sequencing revealed that one of the positive clones selective to RHOA-GTP was different from the RH12. This new RHOA-GTP nanobody is referred to as RH57 (Figure 2A). 

We proceeded with the production and characterization of the binding properties of the RH57 clone in vitro. The nanobody was expressed in *E.coli* periplasm with a carboxy terminal 6xhis-Myc-6xhis fusion or in *E.coli* cytosol with a carboxy terminal Flag-Ctag fusion (see Methods), both fusions including a purification tag and a second epitope tag for further detection (Figure 2B). We focused on the selectivity of the RH57 towards RHOA-GTP by producing and purifying mutants of RHOA that mimic the active and inactive conformations. RHOA L63 is a constitutively active mutant of RHOA (RHOA-CA), which is locked in the GTP-bound conformation, whereas RHOA N19 is considered as a dominant negative one (RHOA-DN). These mutants were expressed with an amino-terminal tag that consists of 2-Strep-Tag^®^ and HA tags. Strep-Tag^®^ was used to capture the GTPase mutants on Strep-Tactin^®^ coated ELISA wells in order to address the RH57 capacity to detect RHOA L63 or N19 mutants. Figure 2C shows a selective detection of RHOA L63 protein in a dose response manner down to 1 nM RH57-Flag-Ctag. This result was consistent with the differential screening on loaded RHOA that led to the identification of the nanobody RH57. As the ELISA sensitivity suggested a relatively high affinity of the RH57 towards RHOA, we evaluated its binding kinetics properties by surface plasmon resonance (SPR). His tagged purified RH57 nanobody was captured on an Ni-NTA chip to perform single cycle kinetics measurements with the two RHOA mutants as analytes or RHOA WT loaded with either GTPγS or GDP. The K_D_ values for RHOA L63 were two-fold lower than for the GTP-bound RHOA WT at 28 nM and 60.5 nM (Figure 2D), respectively, indicating a relatively high affinity of the RH57, but one log lower than the one previously reported for the RH12 [23]. Barely any resonance unit was obtained while RHOA was loaded with GDP, and a low signal was measured with the inactive RHOA N19, allowing an assessment of a K_D_ value above 2 μM, which is a drop of two orders of magnitude compared with the L63 active conformation (Figure 2D). These data demonstrated the high affinity and selectivity of the RH57 nanobody towards the GTP-bound conformation of recombinant RHOA GTPase in vitro, regardless of its production in the oxidative periplasm or in the reducing cytosol of *E.coli*.

### 3.2. Characteriation of the RH57 Intracellular Antibody Binding Properties

Next, we characterized the biochemical properties of the RH57 nanobody when transiently expressed in HeLa cells as an intracellular antibody. For this new molecular construct, we added a carboxy-terminal 6xhis tag for its capture on an IMAC (Nickel ion metal affinity chromatography) matrix followed by a Myc-Tag for detection. We first investigated, by co-precipitation assays, whether the conformational selectivity towards RHO-GTP was preserved while the RH57 was expressed in the intracellular reducing environment. As RHO GTPases are activated at a relatively low basal level in cells cultivated in serum containing medium, we artificially increased the fraction of endogenous proteins in active conformation by loading the cellular extract with GTPγS or GDP as a negative control. We used the GST-RHOTEKIN RBD pulldown, which is the standard biochemical assay to trap the GTP-bound RHO-GTPases, as a positive control. As expected, the RH57 intracellular antibody co-precipitated RHOA only in the GTPγS loaded condition, as did RH12 (Figure 3A). The clear conformational selectivity towards cellular RHOA-GTP led us to perform co-precipitation of the endogenous RHOA subfamily members. The RH57 intracellular antibody captured similar amounts of the active conformation of RHOA/B/C proteins to the RH12 (Figure 3B). To further characterize the binding properties of RH57 intracellular antibody on RHOA protein, we investigated competition with GST-RHOTEKIN RBD/RHOA interaction. Unlike RH12, which totally depleted the RHOA-GTP pull down by the GST-RHOTEKIN RBD, RH57 did not interfere with GST-RHOTEKIN RBD/RHOA interaction (Figure 3C), suggesting that this intracellular antibody did not block endogenous RHO activity inside the cells. Moreover, the RHOA-GTP fraction trapped by the GST-RHOTEKIN RBD in the RH57 expressing cells lysate appeared significantly increased compared with the negative control NR (Figure 3C).

### 3.3. Evaluating the RH57 intracellular antibody as a biosensor of RHOA-GTP. 

**Figure 4:**
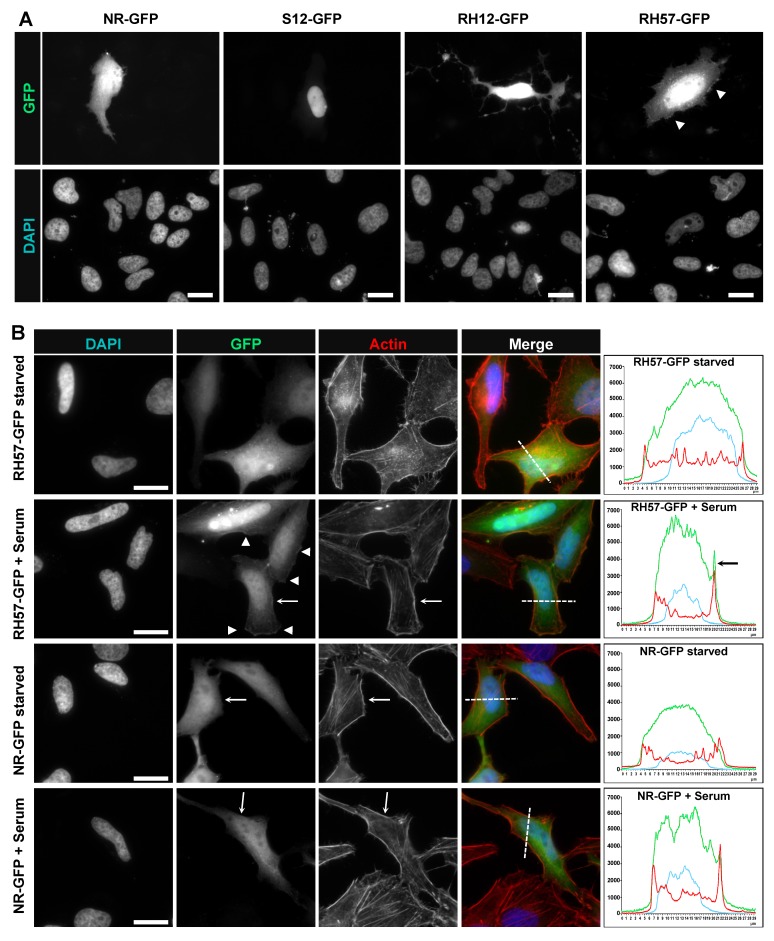
Chromobodies expression in HeLa cells. (**A**) Nanobodies NR, S12 (anti-Histone2A/B), RH12, and RH57 hs2dAb were fused to GFP and expressed in HeLa cells as chromobodies. While RH12 hs2dAb significantly alters cellular shape, potentially by interfering with RHO/RAC pathways, RH57 hs2dAb does not induce such a phenotype on HeLa cells. Arrowheads indicate areas of the plasma membrane with localized increased concentration of the chromobody (enlarged in Figure S1). The scale bars shown in the DAPI panels represent 20 μm. (**B**) HeLa cells transfected with RH57-GFP or NR-GFP and serum starved for 24 h were stimulated with 20% serum to activate RHO GTPases. Arrowheads indicate areas of the plasma membrane with a localized increased concentration of chromobody RH57-GFP upon treatment (enlarged in Figure S2). Cut profile plots display the DAPI (blue), the GFP chromobody (green), and F-Actin (red) intensities corresponding to the dash line shown in the merge channel and arrows in GFP and actin staining channels. F-actin was stained with Alexa 568-Phalloidin. Co-localization of RH57-GFP pixels with F-actin is indicated by large arrows on the plot profile and the channels. Images are representative of transfected cells at moderate expression level. The scale bars shown in the DAPI panels represent 20 μm.

After the initial biochemical validation of the RH57 intracellular antibody, we investigated its behaviour when fused to GFP at its carboxy terminal end. Fluorescent nanobodies, also called chromobodies, have been mainly developed to trace the endogenous localization of the recognized antigen [26,27]. However, the intracellular localization of a chromobody depends on the binding kinetics of the antibody fragment, the expression level of the intracellular antibody as well as its target, and mostly if the target displays a specific localization where it is concentrated enough to re-localize the chromobody. One of the representative cells shown in Figure 4A exemplified the cellular re-localization to the nucleus of the chromatibody S12, which binds Histone H2A/H2B [27]. This condition contrasted with the whole cell staining obtained with the RH57 chromobody or with the non-related negative NR control. Nevertheless, we observed at low confluency that the signals at the cell edge displayed irregular intensities in 60% of the cells expressing the RH57-GFP at a moderate level. Thin areas of higher signal intensity with length of 2 to 5 μm could be observed at the cell periphery (Figure 4A and Figure S1). Such areas were barely detectable in cells expressing the NR-GFP control. The stronger intensity at the cell periphery may reflect membrane dynamics areas where RHO GTPases activity occurs to regulated local actin dynamics in the formation of lamellipodia or membrane ruffles [28]. Moreover, we compared the shape of cells expressing the RH57-GFP chromobody with the one expressing the RH12-GFP. As we have previously reported [23], the RH12 intracellular antibody expression led to a typical phenotype of RHO/RAC inhibition, with impeded acto-myosin dynamics, actin fiber loss, and cell cytoplasm shrinkage, associated with cell death. The cellular shape of cells expressing the RH57-GFP did not induce such dramatic perturbation of the cell even at a high expression level (Figure 4A), indicating that this domain could be a building block to generate a biosensor.

To complete our characterization of the chromobody RH57, we analyzed the signal on fixed HeLa cells transiently transfected, starved for 24 h, and then stimulated with 20% serum for 30 min (Figure 4B). We focused on the fluorescence staining at the edge of the cell and clearly found areas of higher intensity upon serum-mediated RHO stimulation. Lateral cut plot profiles on more than 20 transfected cells showed very heterogeneous pattern of GFP chromobody fluorescence according to the thickness and width of the cells, as well as the expression level in transient transfection. As shown in representative plot profiles (Figure 4B and Figure S2), a peak at one edge of the cell was observed in the profile plot of the stimulated cells expressing the RH57-GFP, whereas the profile of the lateral cut in starved, but not serum stimulated cells displayed a bell-like shape similar to the one found in the NR-GFP control conditions. The peak of RH57-GFP at the cell border co-localized with the cortical actin fibers peak (Figure 4B). This accumulation could correspond to RHO activities, as RHOA-like activity or GEF activities are known to occur close to plasma membrane zones of protrusion or retractions linked to acto-myosin dynamic networks [28,29]. These results suggest that the RH57 intracellular antibody could be used to establish a biosensor of the GTP-bound state of the RHOA-like subfamily.

**Figure 5:**
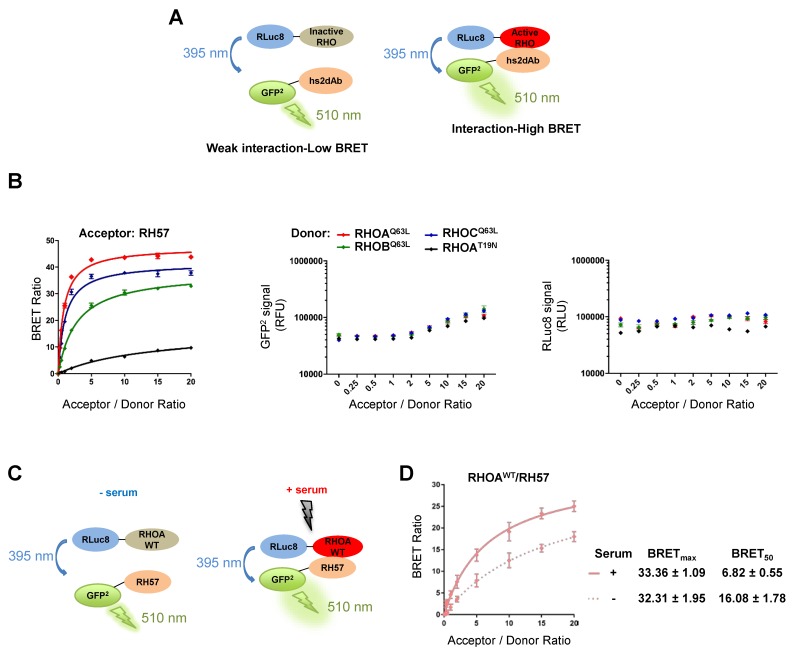
Set up of BRET-based RHO biosensors with anti-RHO hs2dAb to detect RHOA activation. (**A**) Schematic of the BRET2-based RHO biosensor system involving anti-RHO hs2dAb. The full-length RHO proteins are fused at their amino termini to the donor molecule RLuc8. The anti-RHO RH57, selected against the active RHO conformation (RHO-GTP), are fused at their amino termini to the acceptor molecule GFP^2^. When the inactive RHO is expressed with the GFP^2^-hs2dAb, only a weak BRET signal will be detected (left), while when the GFP^2^-hs2dAb interact with the active RHO, they will induce a strong BRET signal (right). The BRET signal or BRET ratio is the ratio between the light emitted at 510 nm divided by the light emitted at 395 nm minus a background correction from the donor only construct. (**B**) BRET donor saturation assays between RHOA^Q63L^, RHOB^Q63L^, RHOC^Q63L^, and RHOA^T19N^ (donors) and the acceptors RH57. Acceptor/donor ratio is the ratio between the quantity of plasmid expressing the acceptor construct divided by the quantity of plasmid expressing the donor construct transfected into HEK293T cells. A ratio of 0 represents the donor only transfected cells condition. Control of the expression of the acceptor (GFP^2^-RH57, GFP^2^ signal) and donors (RLuc8-RHO, RLuc8 signal) from the BRET donor saturation assays are shown. The GFP^2^ level at the acceptor/donor ratio of 0 shows the autofluorescence of the cells [25]. (**C**) Schematic of the BRET2-based RHO biosensor with RH57 to detect RHOA^WT^ activation. Upon serum stimulation, RHOA^WT^ will be activated and interact with RH57. (**D**) BRET donor saturation assay of RHOA^WT^ with RH57. After 20% serum stimulation for 12 min, RH57 contacts RHOA^WT^, as indicated by an increase of the BRET_max_ and a decrease of the BRET_50_ values. The experiments were performed twice (biological repeats) in quadruplicates (technical repeats). Error bars are mean ± SD of the biological repeats.

Finally, we used the RH57 nanobody to produce a BRET biosensor of RHOA activation (Figure 5). We previously developed a BRET-based RAS biosensor toolbox [25] between full-length RAS GTPases and their effectors or binders (such as the anti-RAS intracellular domain antibody, iDAb RAS [20]). Using a similar approach, we have established BRET-based RHO biosensors using the nanobody RH57. The full-length RHO GTPases are fused on their amino terminal end with the donor molecule *Renilla* Luciferase variant 8 (RLuc8). The anti-RHO-GTP binder RH57 is tagged to its amino terminal end with the acceptor molecule GFP^2^. Both donor and acceptor plasmids are transfected into HEK293T cells and if the RLuc8-RHO fusion protein does not interact with the GFP^2^-hs2dAb, the excitation of the donor molecule (RHO) by the luciferase substrate (coelenterazine 400a) will not excite the acceptor fusion molecule (nanobody) (Figure 5A, left panel). However, if an interaction occurs between RLuc8-RHO and a GFP^2^-hs2dAb fusion, bringing the RLuc8 and GFP^2^ within 10 nm, an energy transfer occurs from the RLuc8-RHO donor to the GFP^2^ acceptor and a BRET2 signal is achieved (Figure 5A, right panel). The BRET signal (or BRET ratio) is the ratio between the light emitted by the GFP^2^ acceptor constructs (at 510 nm) upon addition of coelenterazine 400a, divided by the light emitted by the RLuc8 donor constructs (at 395 nm) [30] (see Methods). We performed BRET donor saturation assays to determine whether we could detect an interaction between the RHO and the RH57. If the two proteins interact, the BRET signal will increase and reach saturation, whereas if they do not interact, the BRET signal will increase linearly [31]. Here, RH57 interacts specifically with RHOA, RHOB, and RHOC L63 active mutants, but not with the RHOA N19 dominant negative mutant (Figure 5B), confirming our in vitro data. 

We have implemented the BRET-based biosensor using the RH57 nanobody to detect an activation of RHOA WT upon RHO stimulation with serum (Figure 5C). The serum treatment leads to RHOA activation and to its interaction with RH57 (Figure 5D). Indeed, it largely decreased the BRET_50_ (an approximation of the relative affinity of the acceptor fusion for the donor fusion proteins, corresponding to the acceptor/donor ratio necessary to reach 50% of the BRET_max_) and slightly increased the BRET_max_ (an approximation for the total number of complex RHOA/RH57 and the distance between the donor and the acceptor within the dimer), which together are consistent with an increased affinity of the RH57 nanobody towards RHOA-GTP (Figure 5D). These results suggest that our RHOA^WT^/RH57 biosensor could detect an activation of RHOA in living cells.

## 4. Discussion

Targeting the conformational active state of intracellular proteins requires high affinity and high specificity reagents such as intracellular antibodies. Antibody phage display selection has been useful to isolate very selective intracellular antibodies that discriminate between different cellular pools of a target protein according to its conformational state [32,33,34]. Small RHO GTPase cellular activities rely on such a conformational switch. Their spatio-temporal activation patterns regulate cytoskeleton dynamics and cell morphogenesis in various physiological contexts [35]. Therefore, intracellular antibodies appear as appropriate molecular reagents to develop biosensors of the GTP-bound state of RHO-GTPases, provided that their binding properties are not blocking the cellular function of the target. As a previous selection led to a blocking intracellular antibody that was unsuitable for the production of a sensor, in our current work, we screened the same synthetic nanobody phage display library to identify another intracellular domain antibody that was also selective of a GTP-bound RHOA-like subfamily, but with no apparent inhibitory effect. We demonstrated its high affinity and selectivity in immunoassays and validated its functionality as an intracellular antibody to produce a BRET biosensor of RHO activation.

RHO GTPase activation networks are tightly regulated to avoid sustained activation of morphogenetic signalling pathways [14,15]. Overall, the GTP-bound state is maintained as a minor proportion within the total pool of RHO proteins, which is mostly inactive as judged by biochemical precipitation assays [4,17]. Therefore, the ability of nanobodies to specifically recognize the RHO-GTP target relies on both a strong selectivity towards the GTP-bound conformation of the GTPase and appropriate on- and off-rates of interaction. As the structural superimposition of the two states suggests that the interface of the nanobody interaction should be close to the switch domains, the boundary between a blocking intracellular antibody and a sensing intracellular antibody will depend mainly on their affinity and the epitope overlap with binding domains of natural partners in the intracellular complexity. As the nanobody RH12 was unsuitable for biosensor purposes, we screened the phage display library and identified RH57, which does not compete with the RBD domain, indicating that its binding epitope is unlikely to involve the same interface as the RHO/RH12 interaction. However, we cannot clearly correlate the identification of the RH57 RHO-GTP sensor with any specific modified conditions we have implemented during the phage display selection. For instance, we introduced a depletion step with L63 CA mutants of RHOB and RHOC during the phage display, but we still selected RH12 and the RH57 clones, which are pan-RHO/RAC GTPases antibodies. This indicates that epitopes on the GTP-bound state may not favour the discrimination between RHOs, or that the depletion steps should have been implemented from the first round of selection. 

Chromobodies are powerful tools to trace endogenous antigens within the cell in live cell imaging [19,27]. However, the intracellular localization of a chromobody depends on the binding kinetics of the antibody fragment to the recognized antigen and the expression level of the intracellular antibody as well as its target. Importantly, locally concentrated targets that display a specific localization will give a signal background ratio favourable to distinguish the bound and unbound chromobodies pools. The low expression level of the RHO-GTP state, as well as their transient activation mechanism, may explain the weak signal background ratio of potentially bound to unbound RH57-GFP chromobodies. Although we observed an increased signal at the border of fixed cells, acute sensing and tracking of RHO activities in live cells may require more advanced microscopy approaches dedicated to membrane dynamics imaging such as TIRF (total internal reflction fluorescence) [22,36]. Alternatively, engineering the hs2dAb such that the bound pool displays a shift in fluorescence intensity, such as coupling with solvent sensitive dyes [37], could improve the signal to background ratio. As the RH57 chromobody cannot be easily used as a sensor of endogenous RHO activity for the study of regulators or for the screening of compounds that would modulate RHO activation, we developed a BRET-based RHO biosensor using the RH57 nanobody. BRET-based biosensors have been successfully used to monitor the modulation of interactions with ligands [38,39,40]. Here, we show that the RH57 could be used to detect an activation of RHOA by serum stimulation of the cells. However, the determination of the binding interface of the RH57 on RHOA should be performed by resolving the crystal structure of the complex before using the BRET-based biosensor as a screening platform of ligand/small molecules modulating RHO activation. Indeed, even though the RH57 does not compete with the RBD, we cannot rule out that the RH57 interferes with GAP proteins or other regulators of RHO activity, as we noticed that this intracellular antibody led to an increase of the RHOA-GTP ratio in the GST-ROTHEKIN RBD assay.

In conclusion, we have identified a nanobody from a synthetic library suitable for the production of RHOA subfamily activation biosensors. This genetically encoded molecular tool will allow a broad range of applications to target RHO activity without interfering with RHO signaling. The RH57 domain could also be compared with some RBD used in BRET-based biosensors to monitor RHO activation with a fine spatio-temporal resolution. In the future, the BRET biosensor could allow the screening of small molecules that modulate RHOA/B/C activation. Small molecules could also be selected with this nanobody, adapting analogous methods to those used for intracellular antibody-derived (Abd) compounds targeting RAS [41].

## 5. Patents

L.K., N.B., G.F., and A.O. are co-inventors on the patent PTC/EP2016/052136, concerning the RHO GTP single domain antibodies discovery and their applications.

## Supplementary Materials

The following are available online at http://www.mdpi.com/2073-4468/8/1/8/s1, Figure S1: Chromobodies RH57-GFP expression in HeLa cells, Figure S2: Chromobodies RH57-GFP expression in HeLa cells stimulated with 20% serum after 24h starvation.
