# Supplementary material for: Selection and Characterization of a Nanobody Biosensor of GTP-Bound RHO Activities
Source: Antibodies (Basel). 2019 Jan 9;8(1):8. doi: 10.3390/antib8010008 (PMC6640709; doi:10.3390/antib8010008)
Supplement: Supplementary file 1 [file antibodies-08-00008-s001.pdf]

Supplementary Figures

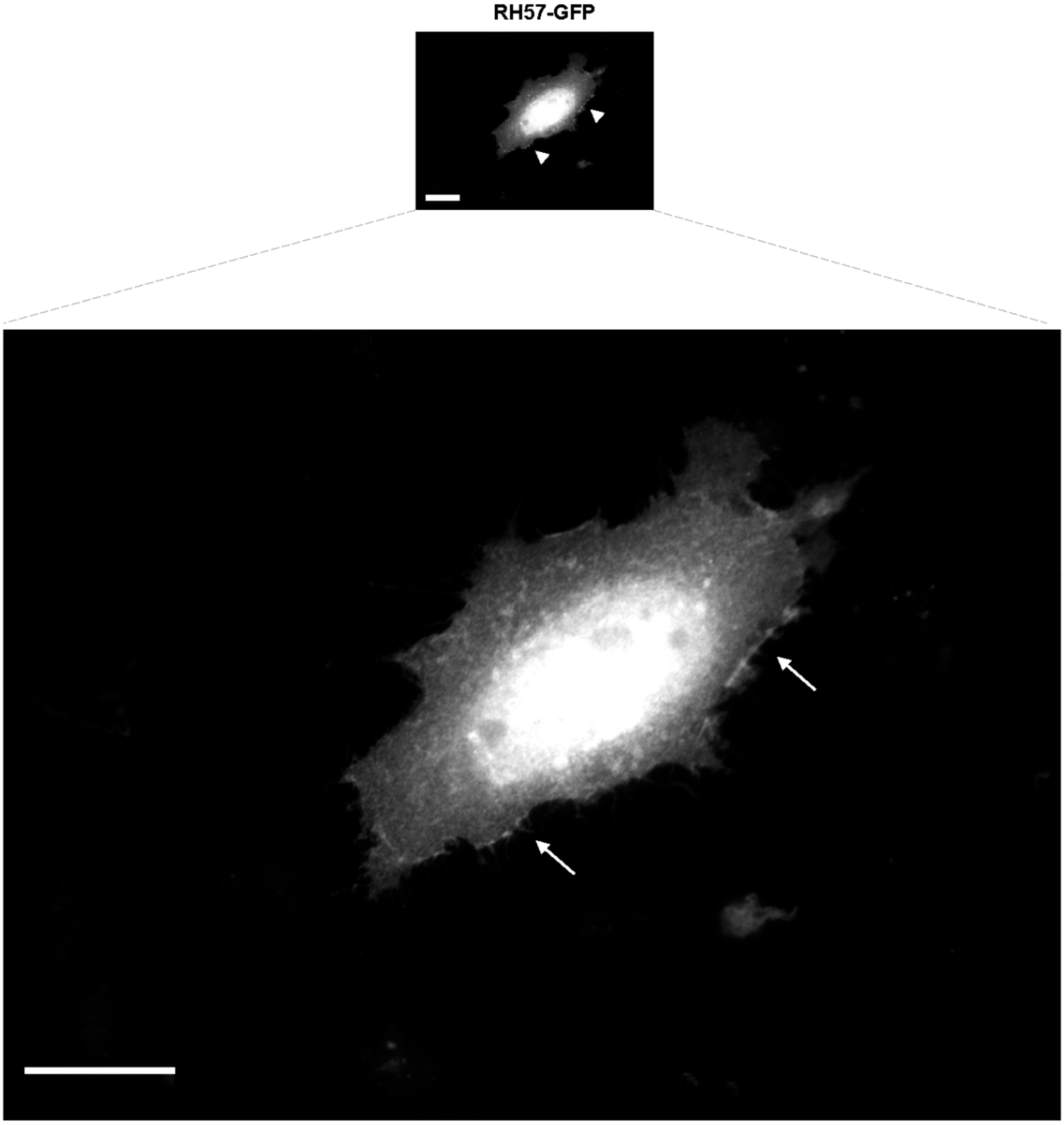

Supplementary Figure 1: Chromobodies RH57-GFP expression in HeLa cells. Higher magnification of the picture shown in Figure 4A in the GFP channel. Arrowheads indicate areas of the plasma membrane with localized increased concentration of the chromobody. The scale bars represent 20 μm.

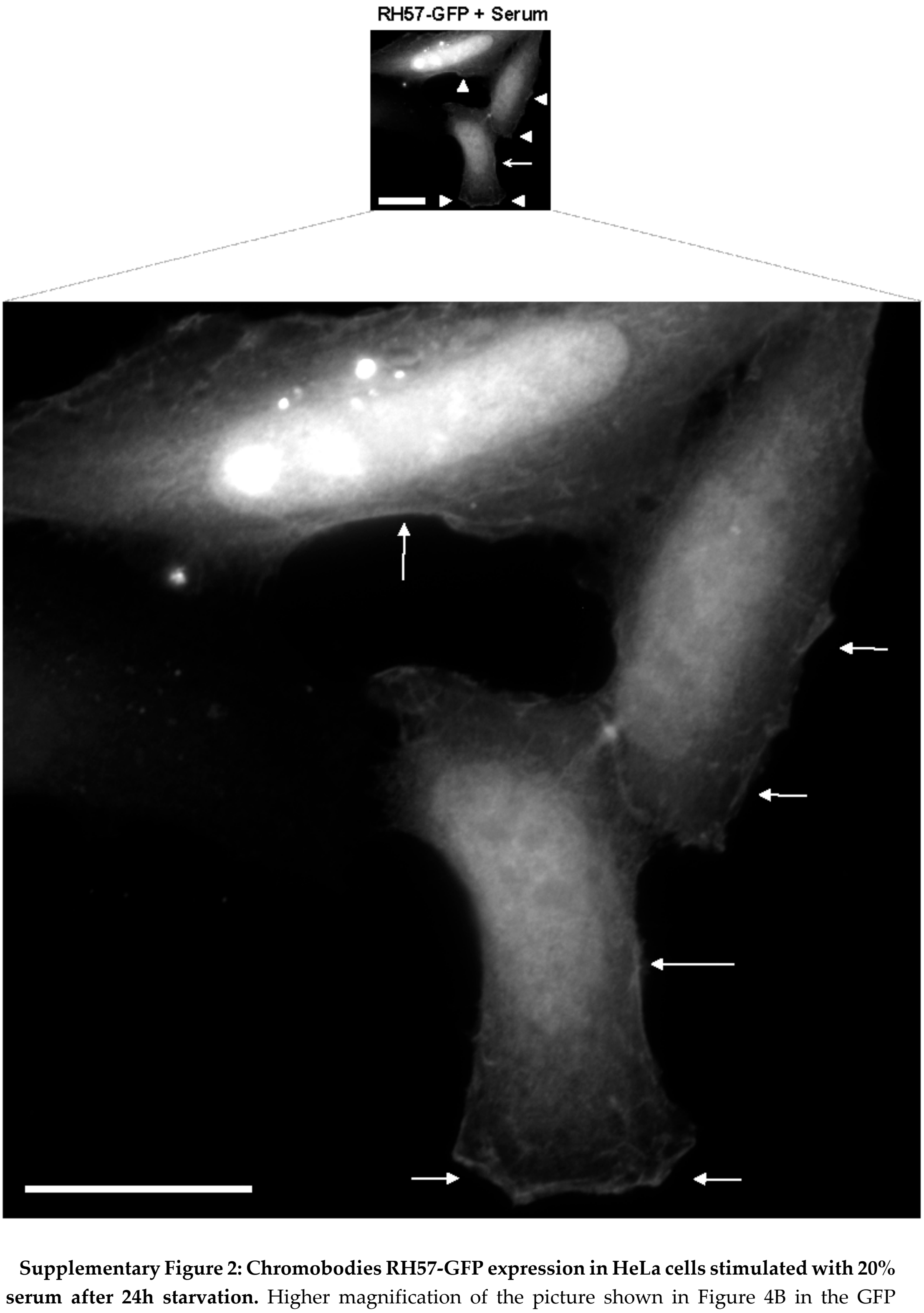

Supplementary Figure 2: Chromobodies RH57-GFP expression in HeLa cells stimulated with 20% serum after 24h starvation. Higher magnification of the picture shown in Figure 4B in the GFP channel. Arrowheads indicate areas of the plasma membrane with localized increased concentration of the chromobody. The scale bars represent 20 μm.
